# Experimental Realization of Sub-THz Circularly Polarized Antenna Based on Metasurface Superstrate at 300 GHz

**DOI:** 10.3390/ma14174796

**Published:** 2021-08-24

**Authors:** Basem Aqlan, Mohamed Himdi, Hamsakutty Vettikalladi, Laurent Le-Coq

**Affiliations:** 1 Electrical Engineering Department, King Saud University, Riyadh 11421, Saudi Arabia; hvettikalladi@ksu.edu.sa; 2 Institut d’Electronique et des Technologies du numeRique (IETR), University of Rennes 1, 35000 Rennes, France; Mohamed.himdi@univ-Rennes1.fr (M.H.); laurent.le-coq@univ-rennes1.fr (L.L.-C.)

**Keywords:** 6G wireless communication, circularly polarized (CP), laser-cutting technology, metasurface (MTS), resonant cavity antenna (RCA), sub-THz

## Abstract

This communication presents a low-profile fully metallic high gain circularly polarized resonant cavity antenna, with a novel single-layer metasurface as superstrate operating at 300 GHz. The unit cell of the metallic metasurface layer consists of perforated grids of hexagonal and octagonal-shaped radiating apertures. The metasurface superstrate layer acts as a polarization convertor from linear-to-circular, which provides left-handed circularly polarized (LHCP) radiation. For simplicity and less design difficulty, a low cost laser cutting brass technology is proposed to design the antenna at sub-terahertz. The proposed circularly polarized resonant cavity antenna prototype has a low-profile planar metallic structure of volume 2.6$\lambda_{0}\times$2.6$\lambda_{0}\times$1.24$\lambda_{0}$. Experimental results validate the design concept. The antenna yields a measured LHCP gain of 16.2 dBic with a directivity of 16.7 dBic at 302 GHz. This proposed circularly polarized resonant cavity antenna finds potential application in 6G sub-terahertz wireless communications.

## 1. Introduction

Nowadays, the sixth generation (6G) wireless systems are a hot topic in academic/industrial research. They are currently designed to operate at higher frequencies, often at the sub-terahertz (THz) spectrum (0.1–1 THz), to increase the throughput of wireless links with features of low-latency [1,2]. The use of the sub-THz frequency band has been established around 300 GHz (252–325 GHz) in IEEE Std.802.15.3d–2017 [3], for future wireless communications. Taking the potential of abundant bandwidths compared to the millimeter wave (MMW) band to achieve short-range connectivity with data rates in the vicinity of 1 Tbps. However, many challenges of high frequency THz communications have to be still addressed. One of the main challenges is molecular absorption and spreading losses are much more noticeable for the THz band compared to lower frequencies. The antenna is a key element for any type of wireless communication systems. Antennas working in this sub-THz frequency band need to be with high-gain characterization, to overcome these drawbacks, and use circular polarization to avoid depolarizing effects, which are big challenges in terms of fabrication processes at higher frequencies. 

THz on-chip antennas are easy to integrate with other circuits, but they also have a lower efficiency and a narrower bandwidth due to the inherent structure of on-chip antennas result and lossy substrate [4,5,6,7,8,9]. In [9], a circularly polarized (CP) substrate-integrated-waveguide (SIW) slot antenna is designed with a simulated gain of −0.5 dBi and a radiation efficiency of 21.4% at 270 GHz. There are a few reported experimentally works on high gain circularly polarized antennas at sub-THz frequencies known in the literature [10,11,12,13,14,15]. A 300 GHz modified Fresnel lens [11], and discrete dielectric lens [12] antennas were manufactured using 3D printing techniques. The volume of lens antennas is oversized, as a result both of them have large profiles, which results in difficulty to process in terms of integration and miniaturization. Another CP sub-THz antenna with conical horn element having a 60 GHz bandwidth was fabricated and tested using a wire electrical discharge machining (EDM) technique [14]. The measured directivity of the horn antenna was 18.3 dBic at 312 GHz. The measured 3 dB axial ratio (AR) of the fabricated antenna prototype had a bandwidth of 7 GHz. However, aforementioned fabricated prototype antennas are bulky in size and have complex structures, especially with other active compact components in integrating with the circuits. Thus, CP planar antennas are required for compact-integrated wireless communication systems at higher frequencies. Antipodal curvedly tapered slot and double-fan-shaped slot antennas operating at 0.5 THz are described in [16,17]. These planar antennas are readily compatible with micromachining techniques, leading to compact-integrated systems at low THz frequencies. Recently, the resonant cavity antennas (RCAs) get more attention among academic researchers due to their planar configuration, high- directivity characteristic and low fabrication difficulty [18,19,20,21,22,23,24,25].

In this communication, we introduce a novel metallic single layer metasurface (MTS), as superstrate, with a resonant cavity antenna (RCA) for high gain with circular polarization at sub-THz. Simulated and experimental results are shown to validate the proposed 300 GHz high-gain fully metallic CP MTS antenna.

## 2. Design Procedure

**Figure 1:**
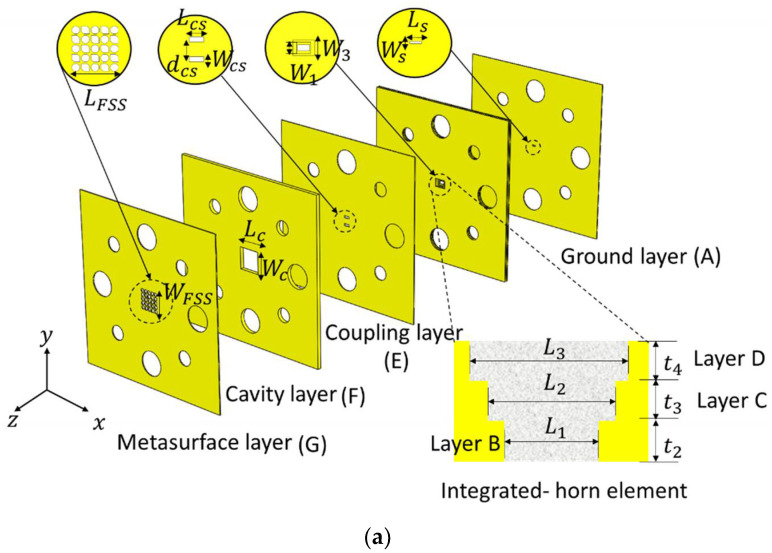

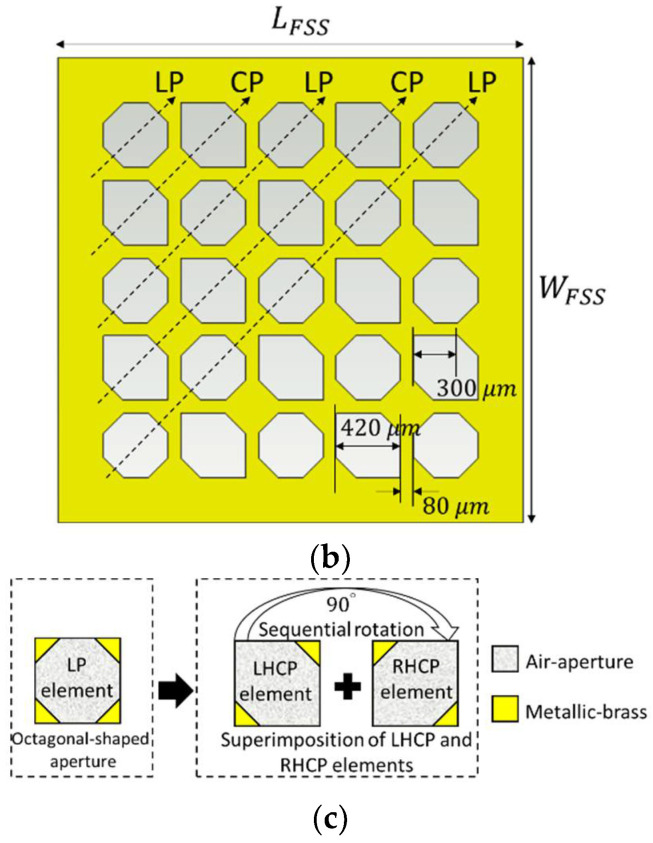
300 GHz CP-RCA: (**a**) exploded model view of 7-layered-structures (brass used for all layers) [25]; (**b**) top view of metasurface layer (dimensions is inset); and (**c**) graphical demonstration of the working principle of the proposed LP octagonal-shaped aperture.

The three dimension (3D) exploded view of the proposed antenna is shown in Figure 1a. The antenna is fed by a standard WM−864 rectangular waveguide (864 × 432 μm^2^). The waveguide is coupled to a ground layer. The ground layer (Layer A) is a slot antenna with 100 μm thickness of metal brass. Layers B, C, and D is an integrated-stepped horn element (three layers) with different thickness of metal brass as shown in Table 1 have been used as multistage to improve the impedance matching bandwidth. Coupling layer (Layer E) consisting of two-parallel slots is used to broaden matching impedance, which are in-phase with ground layer and integrated horn element. Cavity layer (Layer F) is supported metal plate to MTS superstrate layer, having a thickness to achieve resonance condition of Fabry–Perot, which is normally equal to a half-wavelength at resonant frequency of 300 GHz. The basis mathematical analysis used for choosing the dimensions of the proposed antenna structures can be found in [18,19,26], which are calculated by using the simple well-known ray-tracing formula.

The MTS layer is realized by perforated grids of hexagonal and octagonal-shaped radiating apertures made by cutting through a metallic brass layer of finite thickness of 100 μm as presented in Figure 1b. The 5 × 5 array consists of a combination of circularly polarized (CP) elements and linearly polarized (LP) elements. The CP elements are LHCP hexagonal radiating apertures and the LP elements are octagonal-shaped aperture radiators; which is obtained by superimposing one LHCP aperture radiator and one RHCP aperture radiator as shown in Figure 1c. 

For fabrication, we integrate the standard UG−387/U waveguide flange (i.e., alignments and screws holes) into the antenna design. The design parameters detail of layers (units in millimeter) are reported in Table 1.

## 3. Measurements Results and Discussion 

**Figure 2:**
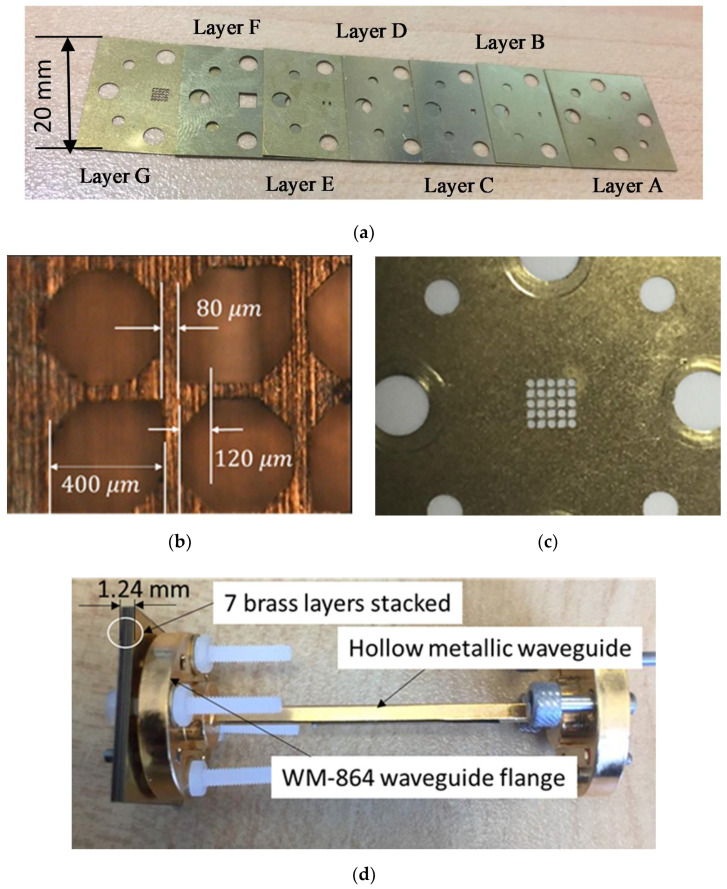
Photographs of manufactured CP-RCA at 300 GHz (**a**) seven metal brass fabricated layers; (**b**) microscopic image of MTS layer; (**c**) enlarged image of CP-MTS layer; and (**d**) fabricated prototype connected to standard UG−387/U waveguide flange [25].

To achieve simplicity in fabrication, each metal layer in the proposed antenna is manufactured by using laser cutting brass technology using a LPKF ProtoLaser U4 laser machine with technical support (Ch. Guitton and F. Boutet) from Manufacturing Measurement Analysis of Radiating Systems (M^2^ARS), Rennes, France. There are seven brass metal layers for one antenna assembly, with different thicknesses as shown in Table 1, and have been used to manufacture the proposed 300 GHz CP-RCA are shown in Figure 2a. All the brass metal layers are fixed by using four plastic screws. The ultraviolet (UV) laser beam of wavelength λ = 355 nm in the UV spectrum is focused on each brass metal layer separately, to obtain different thickness in the desired dimension, with appropriate settings such as a laser cutting speed of 200 mm/s and a laser spot size (i.e., the diameter of the focused laser beam) of 20 µm. 

Using a metallic layer to form a CP-RCA is advantageous in simplifying its fabrication process, based on which the MTS superstrate layer and feeding antenna layers can be fabricated separately then assembled at a later fabrication stage. This reduces the fabrication complexity and cost.

In order to obtain a direct connection to the standard UG−387 waveguide flange, the metal layers contain holes for the alignment with pins and screws without any additional test fixtures or interfaces. This direct-mount technique is superior to the alternative setups using silicon-micromachining without bonding the alignment method [27]. This technology is attractive in terms of low cost and complexity compared with silicon micromachining technology. Additionally, we presented the experimental results showing the proposed 300 CP-RCA characteristics.

Figure 2b demonstrates a microscopic image of the geometry of the novel proposed radiating hexagonal and octagonal shaped aperture unit cells. It is found that the periodicity of the unit cells $p=0.48\;\lambda_{0}$, the unit cell of LHCP unit cell has two symmetrically isosceles triangle chamfers, of $0.12\;\lambda_{0}$, where $\lambda_{0}$ is the operation frequency at 300 GHz. Figure 2c shows an enlarged image of the CP MTS superstrate layer (layer G). For measurements, the proposed antenna must be connected to standard UG−387/U waveguide flange as shown in Figure 2d.

**Figure 3:**
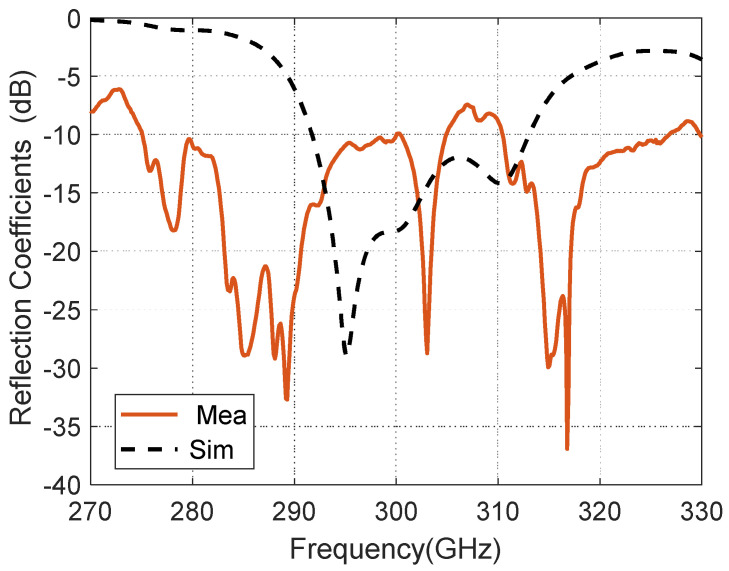
Measured and simulated of the reflection coefficients (S11) of the CP-RCA.

The measured and simulated reflection coefficients (S11) of the proposed CP-RCA are shown in Figure 3. The measured impedance bandwidth for reflection coefficient < −10 dB is 10%, covering from 275 to 305 GHz. There is a discrepancy between the simulated and measured results, which can be attributed to the fabrication tolerances.

**Figure 4:**
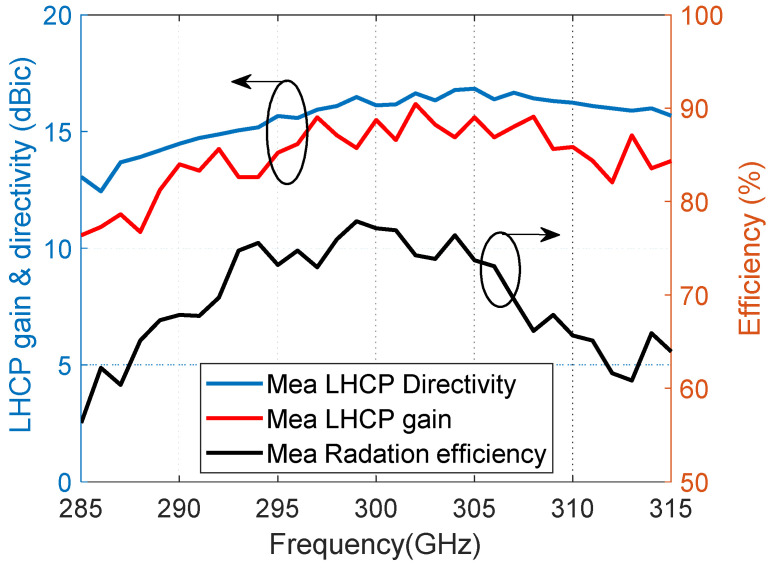
Measured LHCP directivity, realized gain, and radiation efficiency of the proposed antenna.

Figure 4 illustrates the measured LHCP directivity, realized gain and radiation efficiency of the proposed antenna. The maximum LHCP directivity is 16.8 dBic and the maximum LHCP realized gain is 16.2 dBic. The measured overall radiation efficiency is more than 65% from 290 to 310 GHz.

**Figure 5:**
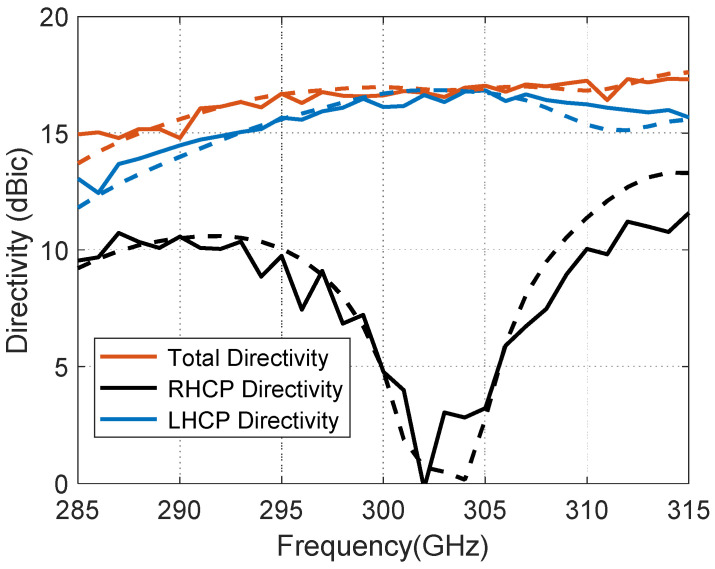
The measured (solid lines) and simulated (dashed lines) of the total, LHCP, and RHCP directivities for the proposed antenna.

The proposed CP-RCA has a measured LHCP directivity of 16.8 dBic and 3-dB LHCP directivity bandwidth of 10% (285–315 GHz) over the desired band, as shown in Figure 5.

**Figure 6:**
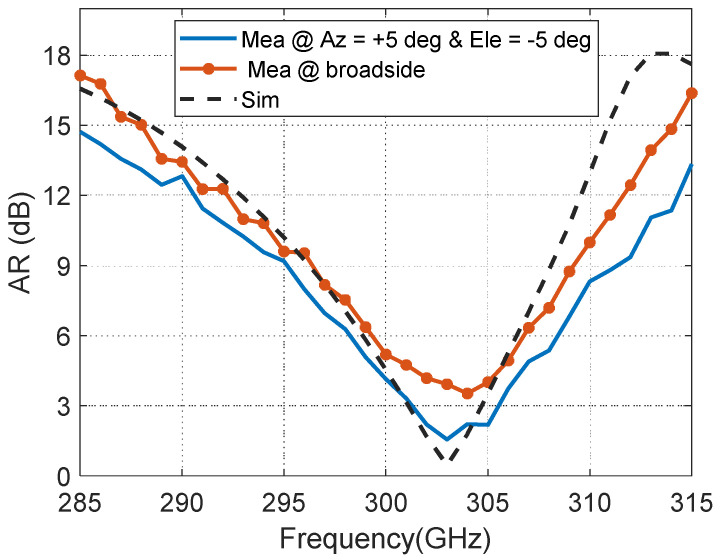
Measured and simulated axial ratio (AR) of the proposed antenna.

It is noted in Figure 6 that, the measured 3-dB AR bandwidth obtained is 4.24 GHz (301.3–305.54 GHz) with a deviation from the broadside direction by 5 degrees in both principal planes due to slight variation in the dimension (the long-side section of hollow waveguide flange Figure 2d), whereas the simulated one is 3.6 GHz (301.1–304.7 GHz).

**Figure 7:**
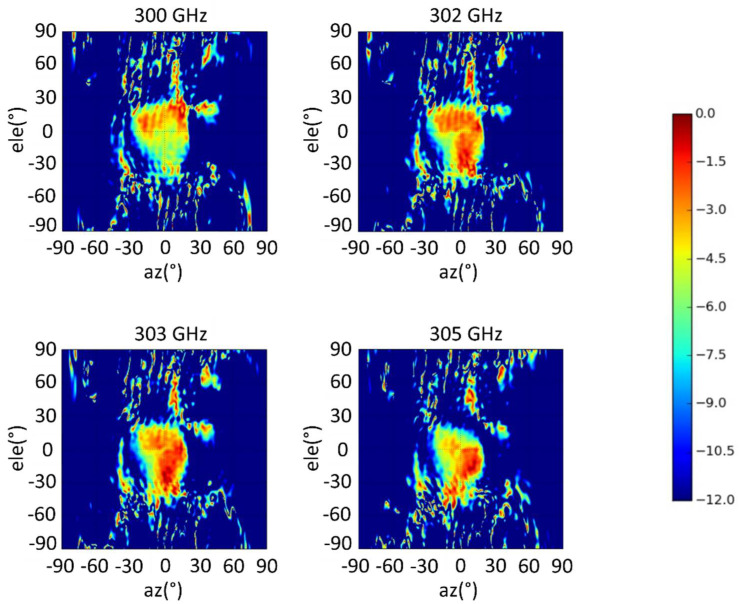
Measured 2D AR of the proposed antenna at different frequencies.

Figure 7 shows the 2D measured AR plots of the proposed antenna at 300, 302, 303 and 305 GHz. The bend of the long-side section of hollow waveguide flange (WM−864 waveguide) is the one that effects broadside direction.

**Figure 8:**
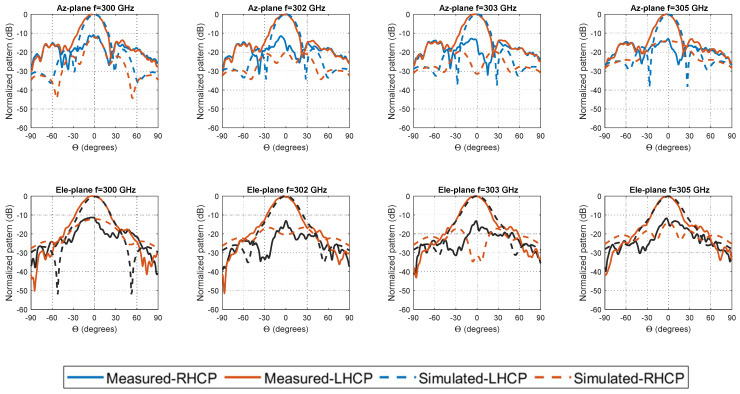
Measured (solid lines) and simulated (dashed lines) radiation pattern of the CP-FPC antenna at 300, 302, 303, and 305 GHz for azimuth (Az.), and elevation (Ele.)-plane cuts.

Figure 8 shows the measured normalized radiation patterns in a compact-antenna test range (CATR) chamber at IETR [28] (funded by the European Union through the European Regional Development Fund, through the CPER Projects 2015–2020 SOPHIE/STIC and Ondes) at 300 GHz, 302 GHz, 303 GHz, and 305 GHz, respectively, for the CP-RCA.

A good agreement is obtained between the measured and simulated far-fields as shown in Figure 8. At higher frequencies (i.e., 300 GHz), the physical dimensions are very tiny, and hence a slight variation in the dimension (the long-side section of hollow waveguide flange Figure 2d has a noticeable effect on the radiation characteristics). The measured results have some deviation in azimuth plane. However, it would be acceptable for such low-cost prototyping fabrication.

**Figure 9:**
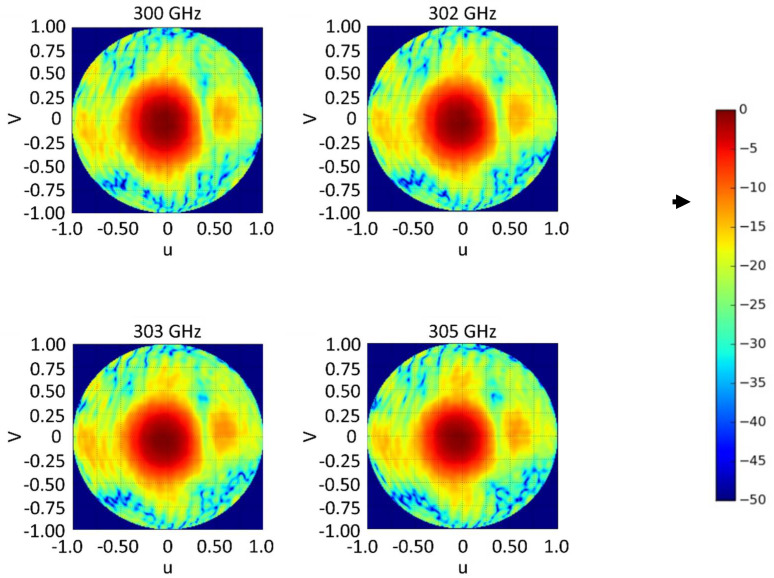
Measured LHCP components of the directivity for the CP-RCA antenna in the u–v spectral plane at different frequencies. The color bar is on the dB scale.

For completeness, the upper hemisphere far-field radiation patterns in the UV-plane at 300, 302, 303, and 305 GHz frequencies, are also plotted in Figure 9. As seen, acceptable side lobe levels (SLLs) of ≤−15 dB is maintained in other azimuthal planes.

Table 2 gives a comparison among different antenna CP in a sub-THz band. A detailed comparison from antenna type, working frequency band, 3 dB AR bandwidth (BW), peak gain (PG), and technology of fabrication is illustrated in Table 2. The results show that our antenna is currently the first one with fully metallic CP high-gain planar structure, which is achieved using simplified laser-cutting brass technology at 300 GHz. The measured results proved that manufacturing parts have a high accuracy.

## 4. Conclusions

A CP-RCA working prototype at 300 GHz band has been presented in this communication. The proposed antenna was designed with the standard WM−864 waveguide flange dimensions, and a laser cutting brass technology is used for the fabrication of different layers in the design. This direct-mount technique is easier to alternative setups of silicon-micromachining, which needs the bonding alignment method and is expensive. On the other hand, silicon micromachining provides more accuracy. 

The proposed antenna yields a measured LHCP gain of 16.2 dBic with a directivity of 16.7 dBic at 302 GHz. A 3 dB measured AR bandwidth of approximately 1.41% (4.24 GHz) with a central frequency of 296 GHz is achieved. The antenna performance is compared to other state-of-art designs. This compact 2.6$\lambda_{0}$ × 2.6$\lambda_{0}$ × 1.24$\lambda_{0}$ antenna can find application in future sub-THz wireless communication systems.

Moreover, it is noted that the above-mentioned RCA radiates LHCP radiation. The RHCP radiation RCA will be generated easily by rotating the MTS superstrate layer by 90°. The new antenna’s simulation results are the same as those for the proposed antenna due to their completely symmetric structure, except that the new antenna radiates RHCP wave. This indicates that both RHCP and LHCP RCAs can be designed with the proposed MTS superstrate layer. 

## Figures and Tables

**Table 1:** Design parameters dimensions (units: mm).

Layers	Param.	Value	Param.	Value	Param.	Value
**Ground**	$t_{1}$	0.1	$L_{s}$	0.46	$w_{s}$	0.1
**Integrated** **horn element**	$t_{2}$	0.1	$L_{1}$	0.8	$w_{1}$	0.4
	$t_{3}$	0.2	$L_{2}$	1	$w_{2}$	0.75
	$t_{4}$	0.2	$L_{3}$	1.42	$w_{3}$	1
**Coupling**	$t_{5}$	0.1	$L_{\mathrm{cs}}$	0.7	$w_{\mathrm{cs}}$	0.25
**Cavity**	$h_{c}$	0.44	$L_{c}$	2.6	$w_{c}$	2.6
**MTS**	$t_{\mathrm{FSS}}$	0.1	$L_{\mathrm{FSS}}$	2.32	$w_{\mathrm{FSS}}$	2.32

**Table 2:** Comparison between the proposed work with other latest CP sub-THz antenna.

Ref.	Antenna Type	Frequency Band	3-dB AR BW (%)	PG (dBic)	Fabrication Technique	Advantage/Disadvantage Structure
[9]	On-chip antenna with SIW	270 GHz	3.29 *	−0.5 *	CMOS 65 nm	Integrated with receiver/Low-gain
[17]	Double-fan-shaped slot	500 GHz	2	12.5	Silicon micromachining	Planar/low-gain
[29]	4 × 4 Slot array	350 GHz	NA	18.4 *	Microfabrication	High-gain/no measured results
[12]	Discrete dielectric lens	300 GHz	18.3	30.8	3D printing	High-gain/bulky
[14]	Conical horn	300 GHz	2.33	18.4	Wire EDM	High-gain/bulky
This work	Resonant cavity–loaded MTS	300 GHz	1.41	16.2	Laser-cutting	Planar and high-gain

* The simulated results, NA (not available).

## Data Availability

Data sharing is not applicable to this article.
